# Enriching plausible new hypothesis generation in PubMed

**DOI:** 10.1371/journal.pone.0180539

**Published:** 2017-07-05

**Authors:** Seung Han Baek, Dahee Lee, Minjoo Kim, Jong Ho Lee, Min Song

**Affiliations:** 1 Institute of Convergence, Yonsei University, Seoul, Korea; 2 Department of Library and Information Science, Yonsei University, Seoul, Korea; 3 Research Center for Silver Science, Institute of Symbiotic Life-TECH, Yonsei University, Seoul, Korea; University of Illinois-Chicago, UNITED STATES

## Abstract

**Background:**

Most of earlier studies in the field of literature-based discovery have adopted Swanson's ABC model that links pieces of knowledge entailed in disjoint literatures. However, the issue concerning their practicability remains to be solved since most of them did not deal with the context surrounding the discovered associations and usually not accompanied with clinical confirmation. In this study, we aim to propose a method that expands and elaborates the existing hypothesis by advanced text mining techniques for capturing contexts. We extend ABC model to allow for multiple B terms with various biological types.

**Results:**

We were able to concretize a specific, metabolite-related hypothesis with abundant contextual information by using the proposed method. Starting from explaining the relationship between lactosylceramide and arterial stiffness, the hypothesis was extended to suggest a potential pathway consisting of lactosylceramide, nitric oxide, malondialdehyde, and arterial stiffness. The experiment by domain experts showed that it is clinically valid.

**Conclusions:**

The proposed method is designed to provide plausible candidates of the concretized hypothesis, which are based on extracted heterogeneous entities and detailed relation information, along with a reliable ranking criterion. Statistical tests collaboratively conducted with biomedical experts provide the validity and practical usefulness of the method unlike previous studies. Applying the proposed method to other cases, it would be helpful for biologists to support the existing hypothesis and easily expect the logical process within it.

## Introduction

Medical informatics has become a fast growing field with the help of a vast amount of biomedical data. Researchers in medical informatics have thrived to make sense of a huge number of academic publications or unstructured data including clinical notes, certain categories of test results such as echocardiograms and radiology reports. Text mining methods were developed for an effective information extraction, knowledge discovery, and hypothesis generation from the literature [1–12]. In the late 80’s, Swanson’s pioneer studies established the foundation for literature-based discovery (LBD) [13,14]. Developments in text mining and hypothesis discovery systems stemming from the early work of Swanson, became coincident with the emergence of conceptual biology. According to the Swanson’s LBD model, when it is known that A term is related to B term and B term is associated with C term in some ways, the implicit relationship between A and C can be suggested as a new plausible hypothesis. With the model Swanson discovered the relationship between Raynaud’s disease and fish oil [13], which was validated through the clinical trial afterward [15]. Later, several studies utilized or extended the Swanson’s model to design discovery systems of better performance or generate new hypotheses [2–6].

**Fig 1:**
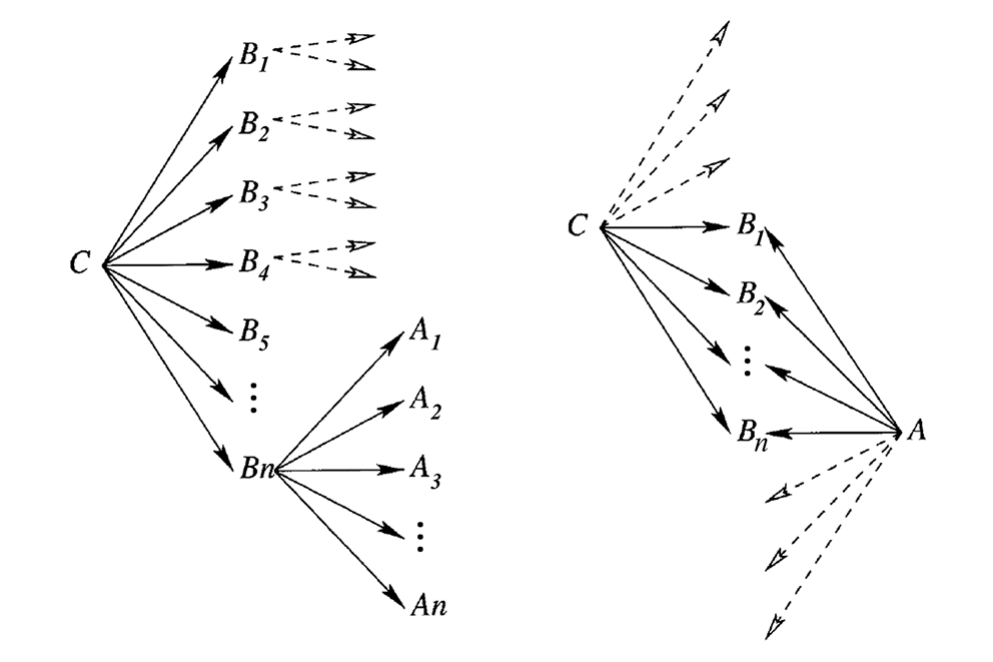
Open (left) and closed (right) discovery process defined by Weeber at al. [16].

Two approaches exist for Swanson’s ABC model (Fig 1) [16]. The process called open discovery begins with A term and try to find C terms that share B terms with A term. In closed discovery process starts from the hypothesis that A term and C term are related to each other, and mines literature to test the hypothesis by searching for the intermediate B terms. Both approaches are frequently used together, in most cases open approach for making a hypothesis and closed one for supporting it.

There were several key limitations found in many of previous works. First, the discovered novel hypotheses were often unconfirmed with a clinical test [4–12]. Such studies stayed as they found the existing facts. Although CoPub Discovery conducted experimental validation for testing their new hypotheses [2], its elicited hypotheses lacked context information and were merely based on the co-occurrence relationship like many other studies. Addressing this point, the proposed technique enriches the plausible new hypotheses with context-based path analysis that allows for storytelling. Storytelling-based path analysis allows for navigation of relation among biological entities that are semantically close to each other besides provision of information about relationship and/or interaction and its directionality. Bell at al. recently stressed the importance of such information while their study did not capture the semantic closeness between entities [7]. Few researchers such as Hristovski et al. began to utilize the semantic relations [8], but their study focuses on hypothesizing a plausible relation between two entities (A- C) and still needs to ensure the practical utility based on the communications with biomedical experts. Our approach along with clinical validation enables to reveal new insights into how a series of entity pairs is organized, and how it can be harnessed for explaining unexpected connections. Thus, we claim that the present paper sheds a new light on hypothesis generation, i.e. hypothesis development and enrichment, discovering a sequential biological pathway (A→B_1_→B_2_··· B_n_→C).

**Fig 2:**
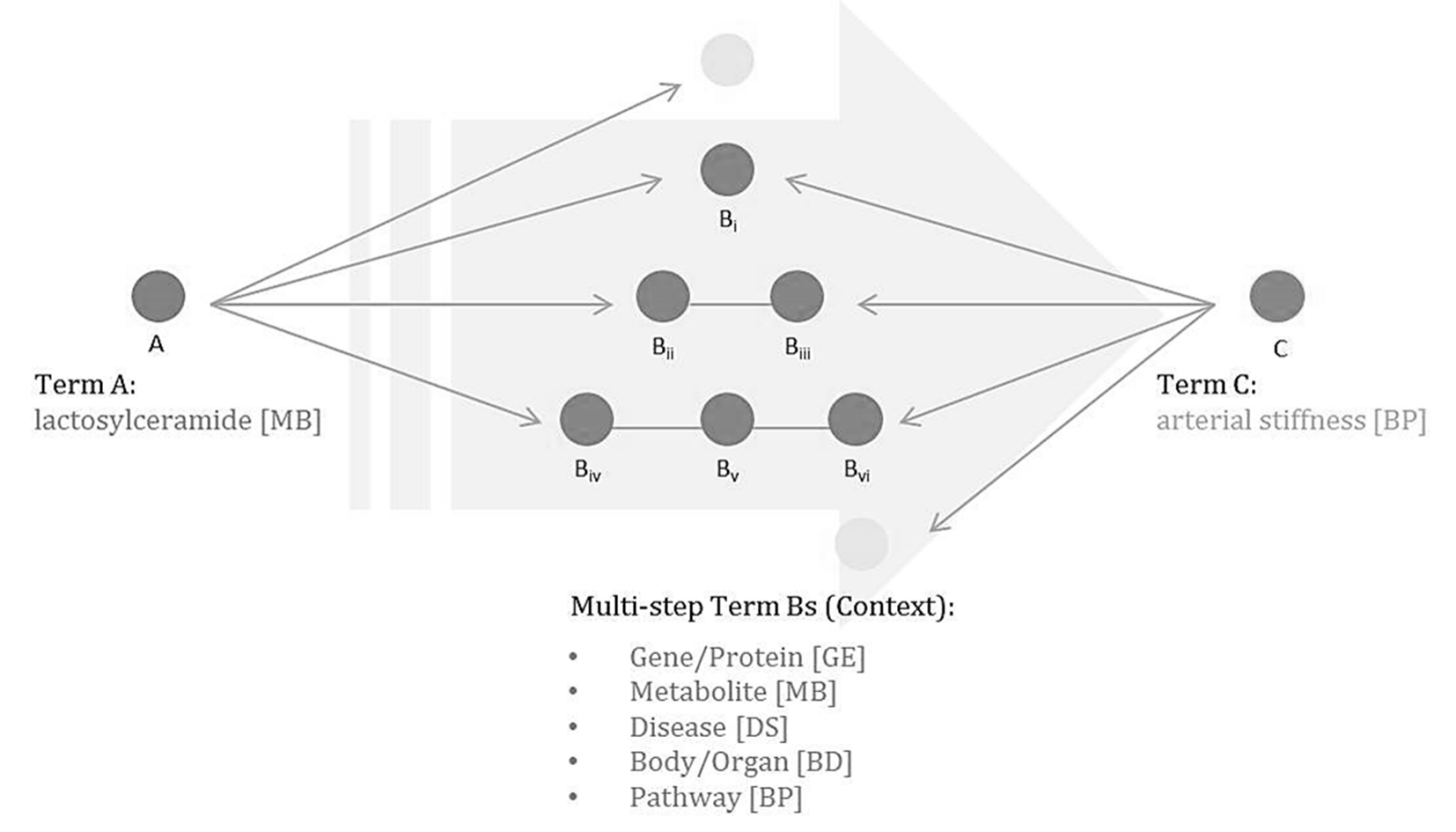
Extension of Swanson’s ABC model.

In this paper, we propose a methodology which would help develop and elaborate their hypotheses based on biomedical text mining and extended Swanson’s model. Specifically, we extend the ABC model of closed discovery to have multiple steps of B terms with various biological types (Fig 2). Starting from the existing hypothesis suggested by the clinical paper, we automatically concretized or elaborated it with a rich context through the experiment. The context was formed by biological entities and relations extracted from the literature. We applied the path finding and ranking algorithm to derive a set of final candidates for the developed hypothesis. The experiment results show that the proposed path finding algorithm identifies plausible hypotheses. One of candidate hypotheses was selected based upon domain experts’ specialized field and knowledge. It is then clinically tested to support the practicability of our proposed method. By the method of context-aware, highly plausible hypothesis enrichment, we wish to make a contribution to the efficient development of the biomedical field.

As a case study, we selected the experimental hypothesis in metabolite research, “An increase in plasma lactosylceramide is an independent predictor of increased arterial stiffness” [17] as the starting point of the experiment. Arterial stiffness is most strongly correlated with cardiovascular disease (CVD) [18] and is measured by brachial-ankle PWV (ba-PWV). Cigarette smoking is known to be a major risk factor for the development and progression of CVD [19, 20]. The effect of smoking on CVD appears, at least in part, by arterial stiffening [21]. Lactosylceramide, one of the ubiquitous glycosphingolipids, is generated in endothelial cells treated with vascular endothelial growth factor, which has been implicated in vascular pathologies [22]. Based on the extended Swanson model (Fig 2), A term is lactosylceramide as a predictor while C term is arterial stiffness as a target predicted entity. B terms can be any biomedical entities which are genes, drugs, or cells, etc. The 5 biological types in our experiment were gene or protein, metabolite, body part of organ, disease, and biological process or pathway. They were selected as appropriate targets to draw meaningful hypotheses with the help of biological experts in the field of metabolite research.

## Methods

### Collection of literature data and resources

We retrieved 911 articles using the query “lactosylceramide” (A term) and 4,845 articles using “arterial stiffness” (C term) from PubMed (http://www.ncbi.nlm.nih.gov/pubmed), a search engine for biomedical literature. After removing one duplicate article, we obtained a total of 5,755 papers in the XML format.

We then constructed five dictionaries, each for one entity type, which are to be used for named entity recognition (NER). The dictionaries are built by collecting names of entities with synonym information from various resources such as ontologies or databases. We restricted resources to human-related ones. Table 1 shows the list of resources we used and the statistics of those integrated dictionaries.

**Table 1:** Resources and statistics of the dictionaries for named entity recognition.

Entity Type	Resources	Number of Unique Entries	Number of Entries including Synonyms
Gene / Protein	EntrezGene [23], UniProt [24]	78,432	289,210
Disease	KEGG Disease [25], Orphanet [26]	10,734	24,605
Metabolite	HMDB [27], KEGG compound [28], Lipid Maps [29], MassBank [30]	80,838	452,273
Body / Organ	Medical Subject Headings (MeSH)	3,616	4,643
Pathway	Gene Ontology [31], KEGG Pathway [25]	27,895	27,934
**Total**	11	201,515	798,665

### Hypothesis development using network-based literature mining

**Fig 3:**
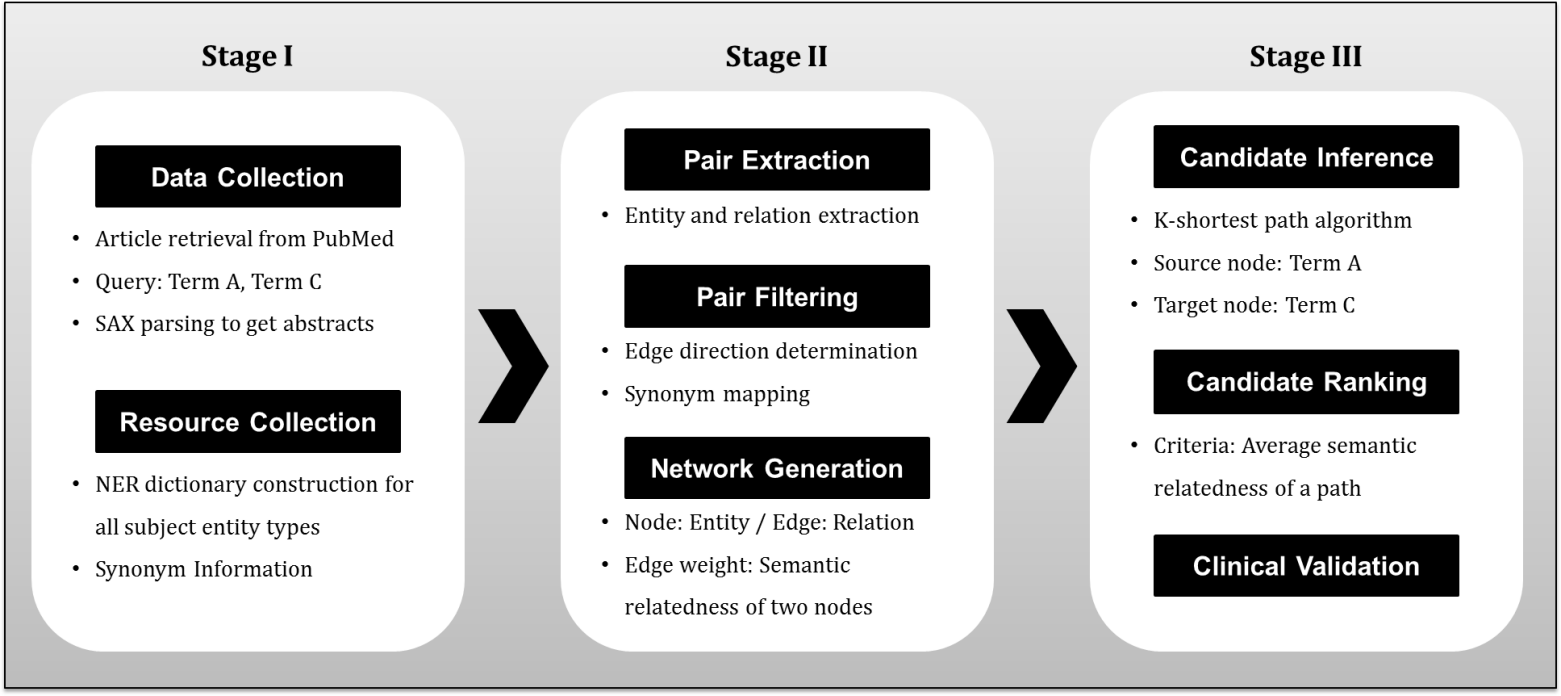
Overview of our proposed approach.

Fig 3 demonstrates our proposed approach. The initial stage involves data and resource collection as described in the preceding sub-section. We gathered 5,755 abstracts from the collected papers with SAX parser, and the five integrated dictionaries of all the subject entity types to apply for entity extraction.

Next, entities and their relations were extracted from the abstracts by using PKDE4J [32]. It enables entity and relation extraction targeting multiple types of entities. It shows a reliable performance over many corpora, achieving average F-measures of 85% for entity extraction and 81% for relation extraction. As it adopts the dictionary-based NER approach, it is able to address almost every biological type if there exist related dictionaries, ontologies, or databases. Its relation extraction (RE) module helps understand the developed hypothesis in contrast with some previous studies using NER only. The dictionary-based or rule-based approach of the two modules has an advantage of overcoming the inflexibility issue of the machine learning approach. In terms of configuration, we set PKDE4J to use abbreviation resolution, lemmatization, string normalization and the five dictionaries we had constructed.

With the result of entity extraction as the input corpus (i.e., the document-entity matrix), we calculated semantic relatedness scores of two entities by the semantic relatedness algorithm [33]. Semantic relatedness in the field of computing refers to how two words are contextually associated with each other, namely the degree of statistically significant co-occurrence of two words. In specific, we adopted the correlated occurrence analogue to lexical semantic (COALS) algorithm [34] for the word space algorithm and the cosine similarity for the final semantic relatedness. The COALS method combined with the cosine similarity is described as:

1. Collect co-occurrence counts (*w*) for context words inside the corpus using windows that are ramped linearly with distance from the target word.
  $$w_{a,b}=the\;number\;of\;co-occurences\;of\;x_{a}\;and\;y_{b}$$
  where *x*_*a*_ means a binary random variable that has value 1 whenever word *a* is the first word chosen and *y*_*b*_ a binary random variable that has value 1 whenever word *b* is the second word chosen.
2. Convert the counts to word pair correlations (*w’*) such that:
  $$w\prime (a,b)=\frac{Tw_{a,b}-\sum_{j}w_{a,j}∙\sum_{i}w_{i,b}}{\sqrt{\left(\sum_{j}w_{a,j}∙(T-\sum_{j}w_{a,j})∙\sum_{i}w_{i,b}∙(T-\sum_{i}w_{i,b})\right)}}$$
  where
  $$T=\sum_{i}\sum_{j}w_{i,j}$$
  Set negative correlation values to 0 and take square roots of the remaining (positive) values.
3. Calculate the semantic relatedness (*s*) between two words using the cosine similarity of the words’ vectors (*a* and *b*):
  $$s(a,b)=\frac{\sum_{i}a_{i}\times b_{i}}{\sqrt{\sum_{i}{\left(a_{i}\right)}^{2}}\times \sqrt{\sum_{i}{\left(b_{i}\right)}^{2}}}$$

We also detect voice of relation so that the direction of relations in a passive voice relation is determined as one from right-side entity to left-side entity, otherwise generally from left-side entity to right-side entity. After generating directed pairs with the extracted entities and relations, we carried out synonym mapping to aggregate entities with the same meaning and similar relations. Synonyms were compiled by the five NER dictionaries and the bio-verb list created by PKDE4J. The overlapping pairs caused by synonym mapping were then unified. With the final entity pairs, we generated a directed network where the entities and relations became nodes and edges respectively. The edges were weighted by the corresponding semantic relatedness score.

In the final stage, we extracted the directed paths from the constructed network―in other words, candidates for the developed hypothesis―from the directed network. For path finding, we applied Yen’s *k*-shortest path algorithm [35] and it can be illustrated as:

1. Consider a directed graph with *N* nodes *v*_*1*_, *v*_*2*_, …, *v*_*n*_ and *M* edges.
2. Initialize heap *B* to store the potential *k*^*th*^ shortest path between a source node (*v*_*1*_), and a target node (*v*_*n*_).
3. Determine *A*^*1*^ as the shortest path using Dijkstra’s algorithm [36] where *A*^*k*^ is the *k*^*th*^ shortest path from *v*_1_ to *v*_n_.
4. Iterate *k* − 1 times to determine *A*^*i*^, *i* = 2, 3, …, *k*.
  - Find all deviations of *A*^*i−1*^ and add each one to heap *B*.
  - Extract the minimum cost path from heap *B* as *A*^*i*^.

Using the algorithm, we acquired 91 candidate hypotheses. They are further ranked by each path’s average semantic relatedness score as we consider it as a path’s expected reliability score, assuming that having a high average semantic relatedness implies involving entities which tend to be closely related to each other.

To examine whether generated hypotheses can be developed to be clinically meaningful (in which the extracted entities are more likely to be connected with each other in a biological point of view), two biomedical experts of metabolite research reviewed the candidate list. Since our work focuses on concretizing the hypothesis with multiple B terms, we got rid of all the candidates with only one B term. The list was then filtered to keep hypotheses with the specific types of B terms which corresponded to their area of expertise (i.e. metabolites), in order to ensure the feasibility of the clinical experiment to be done by them immediately. They eventually chose the second-ranked hypothesis in the final list as they judged it based on their domain knowledge, expecting its high possibility of having biological significance.

For testing the selected hypothesis, they included 56 male subjects aged under 50 years in the clinical test, and divided them into smoker or non-smoker group. All subjects provided written informed consent before participation in this study, which was approved by the Institutional Review Board of Yonsei University and complied with the Declaration of Helsinki. Biochemical characteristics were analyzed and statistical analysis has been done to see if the new hypothesis automatically elaborated by biomedical literature mining is meaningful. The methods and procedures for laboratory experiments and statistical analysis have been described in detail in a previous study [17].

## Results

### Result of biomedical literature mining

**Fig 4:**
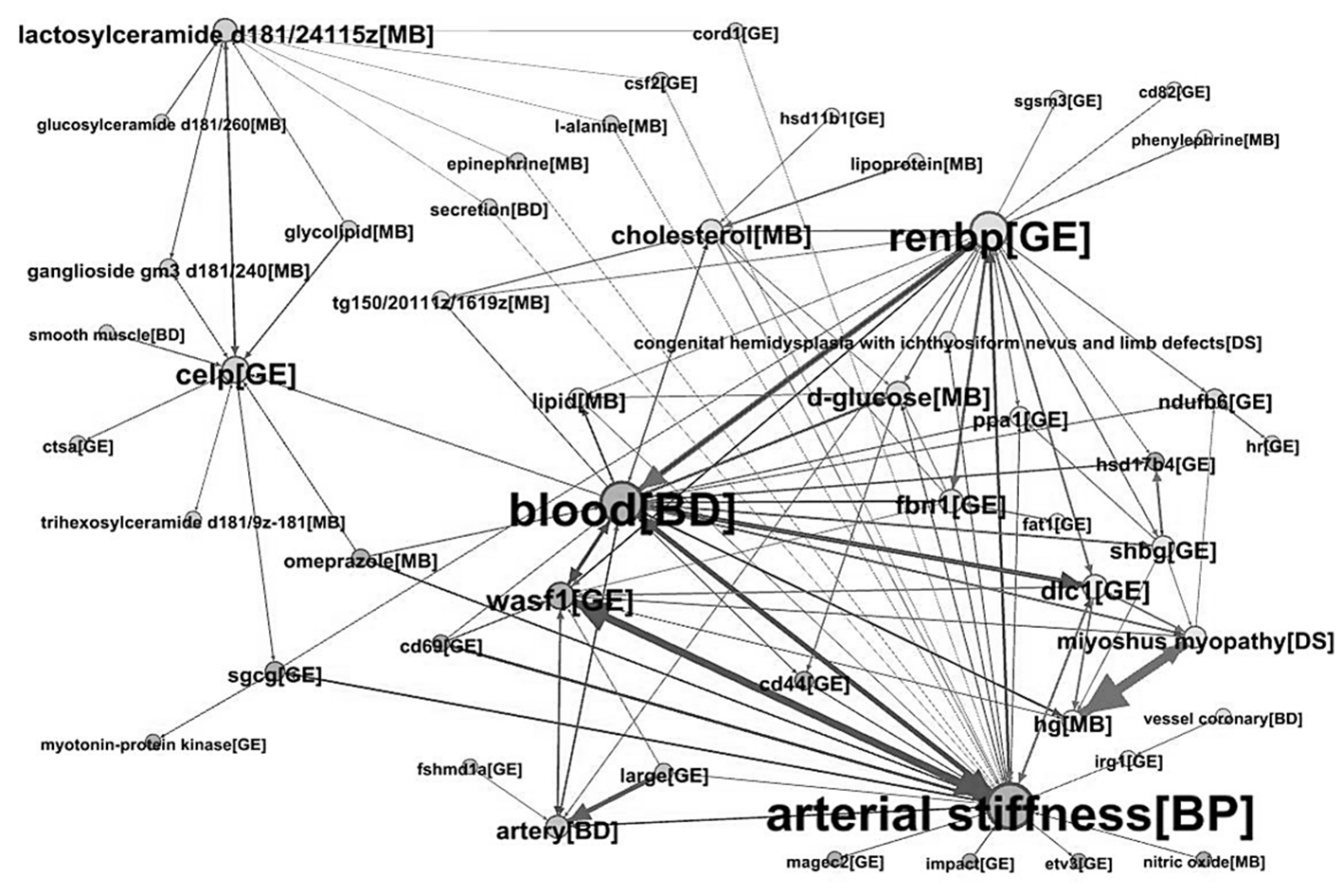
Visualization of a portion of the directed network generated by literature mining.

Through our proposed methodology, we constructed the directed network that contains a potential explanation of how the change in lactosylceramide is related with the change in arterial stiffness by showing B terms between them (Fig 4). The number of nodes (entities) was 2,763 and that of edges (relations) was 32,493. Among 2,763 unique entities, 1,383 of them were genes or proteins, 878 metabolites, 227 diseases, 210 biological processes or pathways, and 65 body parts or organs. The most frequently co-occurring relationship was the one between age and blood, implying that they are closely related and they are key factors when discussing the association of lactosylcermide and arterial stiffness.

We derived a total of 91 candidates for the developed hypothesis from the network. Table 2 presents top ranked ones that seem to have the most credibility. Each of them shows a unique and possible path where lactosylceramide is likely to affect arterial stiffness. For example, the candidate with the second highest semantic relatedness score enables storytelling on the starting hypothesis as follows. The *Contain* relationship between lactosylceramide and inulobiose may be able to affect 4-aminohippuric acid, followed by the impact on arterial stiffness. The supporting information about such relationships among two of those entities constructing a path can be found in previous studies [37,38] since we allow our experiment to track papers which have descriptions about the extracted biomedical relationships between two entities by using PMID, an identifier of PubMed articles.

**Table 2:** Top ranked candidates of the developed hypothesis.

Rank	A term	Relation (→)	1^st^ B term	Relation (→)	2^nd^ B term	Relation (→)	3^rd^ B term	Relation (→)	C term	Average Semantic Relatedness
1	LacCer (MB)	Co-occur	MGAM (GE)	Transmit	Equol (MB)	Co-occur	Daidzein (MB)	Co-occur	Arterial Stiffness (BP)	0.209499
2	LacCer (MB)	Contain	Inulobiose (MB)	Co-occur	4-aminohippuric acid (MB)	Co-occur			Arterial Stiffness (BP)	0.143843667
3	LacCer (MB)	Co-occur	MGAM (GE)	Co-occur	Rosuvastatin (MB)	Co-occur	3-nitrotyrosine (MB)	Co-occur	Arterial Stiffness (BP)	0.104826
4	LacCer (MB)	Method	FLT3LG (GE)	Method	-	-	-	-	Arterial Stiffness (BP)	0.060555
5	LacCer (MB)	Contain	Inulobiose (MB)	Co-occur	-	-	-	-	Arterial Stiffness (BP)	0.059905
6	LacCer (MB)	Co-occur	MGAM (GE)	Co-occur	Doxazosin (MB)	Report Co-occur	-	-	Arterial Stiffness (BP)	0.044014333
7	LacCer (MB)	Co-occur	MGAM (GE)	Co-occur	Lipoamide (MB)	Co-occur	-	-	Arterial Stiffness (BP)	0.041227333
8	LacCer (MB)	Report	Breast cancer (DS)	Co-occur	ATP8A2 (GE)	Co-occur	-	-	Arterial Stiffness (BP)	0.039473333
9	LacCer (MB)	Co-occur	MGAM (GE)	Report	Inulobiose (MB)	Co-occur	-	-	Arterial Stiffness (BP)	0.039037333
10	LacCer (MB)	Co-occur	FBF1 (GE)	Co-occur	Atrial fibrillation (DS)	Co-occur	KIAA0101 (GE)	Modify	Arterial Stiffness (BP)	0.035331
11	LacCer (MB)	Report	Tay-sachs disease (DS)	Report Co-occur	-	-	-	-	Arterial Stiffness (BP)	0.03115
12	LacCer (MB)	Co-occur	ENG (GE)	Report	Malondialdehyde (MB)	Co-occur	-	-	Arterial Stiffness (BP)	0.027345333
13	LacCer (MB)	Co-occur	SEPT5 (GE)	Plain	POMT1 (GE)	Co-occur	SLC26A3 (GE)	Increase	Arterial Stiffness (BP)	0.027308
14	LacCer (MB)	Co-occur	Nitric Oxide (MB)	Co-occur	Malondialdehyde (MB)	Co-occur	-	-	Arterial Stiffness (BP)	0.026963333
15	LacCer (MB)	Co-occur	CAMK4 (GE)	Co-occur	FN1 (GE)	Co-occur	-	-	Arterial Stiffness (BP)	0.026768
16	LacCer (MB)	Co-occur	MGAM (GE)	Co-occur	ATP8A2 (GE)	Co-occur	-	-	Arterial Stiffness (BP)	0.025134333
17	LacCer (MB)	Co-occur	Propyl Gallate (MB)	Co-occur	ATP8A2 (GE)	Co-occur	-	-	Arterial Stiffness (BP)	0.025118667
18	LacCer (MB)	Co-occur	Acrylamide (MB)	Report	CAMK4 (GE)	Co-occur	FN1 (GE)	Co-occur	Arterial Stiffness (BP)	0.0242185
19	LacCer (MB)	Plain	ATN1 (GE)	Co-occur	CISH (GE)	Report	-	-	Arterial Stiffness (BP)	0.021235
20	LacCer (MB)	Co-occur	ABCB1 (GE)	Increase	ELOVL6 (GE)	Co-occur	-	-	Arterial Stiffness (BP)	0.021224
21	LacCer (MB)	Increase Modify	fut4[GE]	Co-occur	HMGB1 (GE)	Modify	-	-	Arterial Stiffness (BP)	0.019730333
22	LacCer (MB)	Increase	Phosphate (MB)	Co-occur	Folic acid (MB)	Decrease	-	-	Arterial Stiffness (BP)	0.019715
23	LacCer (MB)	Co-occur	FBF1 (GE)	Modify	ETV3 (GE)	Co-occur	ATP8A2 (GE)	Co-occur	Arterial Stiffness (BP)	0.01826025
24	LacCer (MB)	Increase	LPA (GE)	Co-occur	-	-	-	-	Arterial Stiffness (BP)	0.0169255
25	LacCer (MB)	Increase	DYM (GE)	Modify	-	-	-	-	Arterial Stiffness (BP)	0.015764

In the *Entity* columns, square brackets contain the biological type information of the corresponding entity. (MB): Metabolite, (GE): Gene/Protein, (BP): Biological Process/Pathway, (DS): Disease, and (BD): Body/Organ. LacCer is the acronym for lactosylceramide.

### Performance evaluation with BITOLA and SemRep

We have used BITOLA a biomedical discovery support system created by Dimitar Hristovski and Borut Peterlin, to examine and compare our results [39]. We examined whether the top 25 paths that the proposed approach discovered, is also able to be connected when using BITOLA. As shown in S1 Table, while most of our B terms did not directly connect to each other or with A and C terms, they were connected through intermediated B terms between them. We added the frequency between XY (while X = A or B terms and Y = intermediate B terms) and YZ (while Y = intermediate B terms and Z = B or C terms) for each intermediate B terms, and added the sum to obtain the value of XZ (while X = A or B terms and Z = B or C terms). However, though the number of B terms could be over 200, because BITOLA interface only shows maximum 200 B terms, we were only able to obtain XZ values for 200 B terms when the number of B terms was over 200. While the frequency of ‘directly’ connected nodes was not tuned, we divided the ‘indirect’ values with ‘2’ to give penalty, and ranked them by their total.

As shown in S1 Table, while some of our paths were not connected fully because some of our entities were not recognized by BITOLA, most of our paths were connected fully through intermediate B terms. Though, ‘directness’ and the ‘frequency’ that are given by BITOLA show the ‘co-occurrence’ relatedness between two entities, in which ‘directness’ indicates whether the two entities co-occur directly or indirectly and the ‘frequency’ shows how often, it does not signify that the two entities are related in a biological point of view. Although it is true that our paths are not truly directional when relations such as "contain" or "co-occur" or "method" is extracted between two entities, our system is able to show ‘direction’ when relation between two entities shows causality. As the frequency that is given by BITOLA is based on co-occurrence frequency between two entities, it is logical comparing the values, in which higher frequency indicates that the two entities are closely related by co-occurrence. However, this gives difficulties when comparing between ‘direct’ and ‘indirect’ connections, in which ‘direct’ connections should be considered more valuable. Comparing the values between ‘direct’ and ‘indirect’, without tuning them, is most likely to result in lower values for ‘direct’ connections which in the end results in lower ranking of directly connected paths.

We also used SemRep to compare our results [40]. Because our focus was finding B terms between the two entities we fixed the A term to ‘lactosylceramide’ and the C term to ‘arterial stiffness’, and extracted 25 paths as shown in S2 Table. While it did show the relation between two entities, it also mostly consists of relations that do not show causality. Among the extracted 25 paths we were able to find new B terms such as ‘NOS3 (Nitric Oxide Synthase 3) protein’, which plays a role in nitric oxide (NO) production. Though our system shows ‘nitric oxide’ as one of the B terms, NOS3 protein itself would be an interesting candidate B term connecting lactosylceramide and arterial stiffness. We also found ‘aging’ a biological process as a new B term. However ‘aging’ was found in a repeating pattern with in a single path, such as ‘rank 1’ shown in S2 Table. Although indeed lactosylceramide, aging, and arterial stiffness are closely related, it is difficult to comprehend the whole path due to such repeating B terms. Similar to our system we observed entities such as ‘cell’ in which the concept is too broad to interpret with other connected entities.

### The web-based system for the proposed approach

We provide our system on the following URL: http://informatics.yonsei.ac.kr:8080/hypothesis_generator/index.html

A brief instruction for our system is as follows.

1. Type in a search term or type in multiple search terms that you want to search.
2. The search terms or search term you have entered will be highlighted within the result. PubMed ID for each result will be shown on the left and a direct link to the article is given in the right. You are able to choose the number of PubMed records to be included for generating the paths.
3. Type in the entities that you want to path analysis from the list of entity names. The left will be the ‘A-term’ and the right will be the ‘C-term’ of your path. You are able to choose the number of path you want to analysis as shown.
4. The results will be shown after clicking on the ‘Path Analysis’. Relation between the entities are shown in the brackets.

### Performance evaluation of the proposed methodology

The list of Table 2 was filtered to become Table 3 in a way described in the Material and Methods section. Among all six hypotheses shown in Table 3, hypothesis with the 2^nd^ ranking score was the only hypothesis that was fully ‘directly’ connected when using BITOLA while SemRep did not show any matching hypothesis. This indicates that the entities consisting the path are highly related by ‘co-occurrence’. Indeed our experts also chose the candidate hypothesis with the 2^nd^ ranking score among all six hypothesis candidates in that the entities that constitute the path were more likely to be connected (in a biological context matter) with each other. Therefore, the second ranked hypothesis was considered as more meaningful than others in a biological point of view, while in other hypothesis candidates, the extracted relations among entities or due to the nature of the extracted entities itself were difficult to interpret. For example in 1^st^ ranking score hypothesis, lactosylceramide is connected to Inulobiose through the relation ‘contain’ which is difficult to comprehend. In addition, although it is true inulobiose, 4-aminohippuric acid and arterial stiffness co-occur with each other, the relation between them are not clear because changes in arterial stiffness is independent of inulin and p-aminohippuric acid(synonym for 4-aminohippuric acid) clearance [41]. While in 4 paths (from the third rank to sixth rank hypothesis), the entities such as ‘phosphate’, ‘sucrose’ and ‘phospholipid’ are too broad to interpret in a meaningful manner in relation to other connected entities.

**Table 3:** Top ranked candidates with multiple B terms of metabolites.

Rank	A term	Relation (→)	1^st^ B term	Relation (→)	2^nd^ B term	Relation (→)	3^rd^ B term	Relation (→)	C term	Average Semantic Relatedness
1	LacCer (MB)	Contain	Inulobiose (MB)	Co-occur	4-Aminohippuric acid (MB)	Co-occur	-	-	Arterial Stiffness (BP)	0.1438
2	LacCer (MB)	Co-occur	Nitric Oxide (MB)	Co-occur	Malondialdehyde (MB)	Increase	-	-	Arterial Stiffness (BP)	0.0270
3	LacCer (MB)	Increase	Phosphate (MB)	Co-occur	Folic acid (MB)	Decrease	-	-	Arterial Stiffness (BP)	0.0197
4	LacCer (MB)	Increase	Phosphate (MB)	Co-occur	Hydrogen carbonate (MB)	Contain	Sucrose (MB)	Co-occur	Arterial Stiffness (BP)	0.0036
5	LacCer (MB)	Plain	Silicon (MB)	Plain	Hydrogen carbonate (MB)	Contain	Sucrose (MB)	Co-occur	Arterial Stiffness (BP)	-0.0021
6	LacCer (MB)	Increase	LacCer surfate (MB)	Plain	Phospholipid (MB)	Report	-	-	Arterial Stiffness (BP)	-0.0034

In the *Entity* columns, square brackets contain the biological type information of the corresponding entity. (MB): Metabolite, (GE): Gene/Protein, (BP): Biological Process/Pathway, (DS): Disease, and (BD): Body/Organ. LacCer is the acronym for lactosylceramide. ‘Plain’ describes relations that did not have causality nor can be classified by our category, due to the ‘verb’ extracted between two entities have no causality nor can it be classified by our category. While, ‘co-occurrence’ a ‘verb’ does not exist to describe the relation.

The 2^nd^ ranking score hypothesis suggests a potential pathway that consists of lactosylceramide, nitric oxide (NO), malondialdehyde (MDA), and arterial stiffness. To test the chosen hypothesis, we performed a clinical experiment on 22 smokers and 24 non-smokers, a total of 46 male subjects under 50 years old. Due to ‘laboratory conditions’ and ‘experimental limitations’ of our experts who mainly focus on metabolic changes, we were not able to evaluate other paths through laboratory experiments.

**Fig 5:**
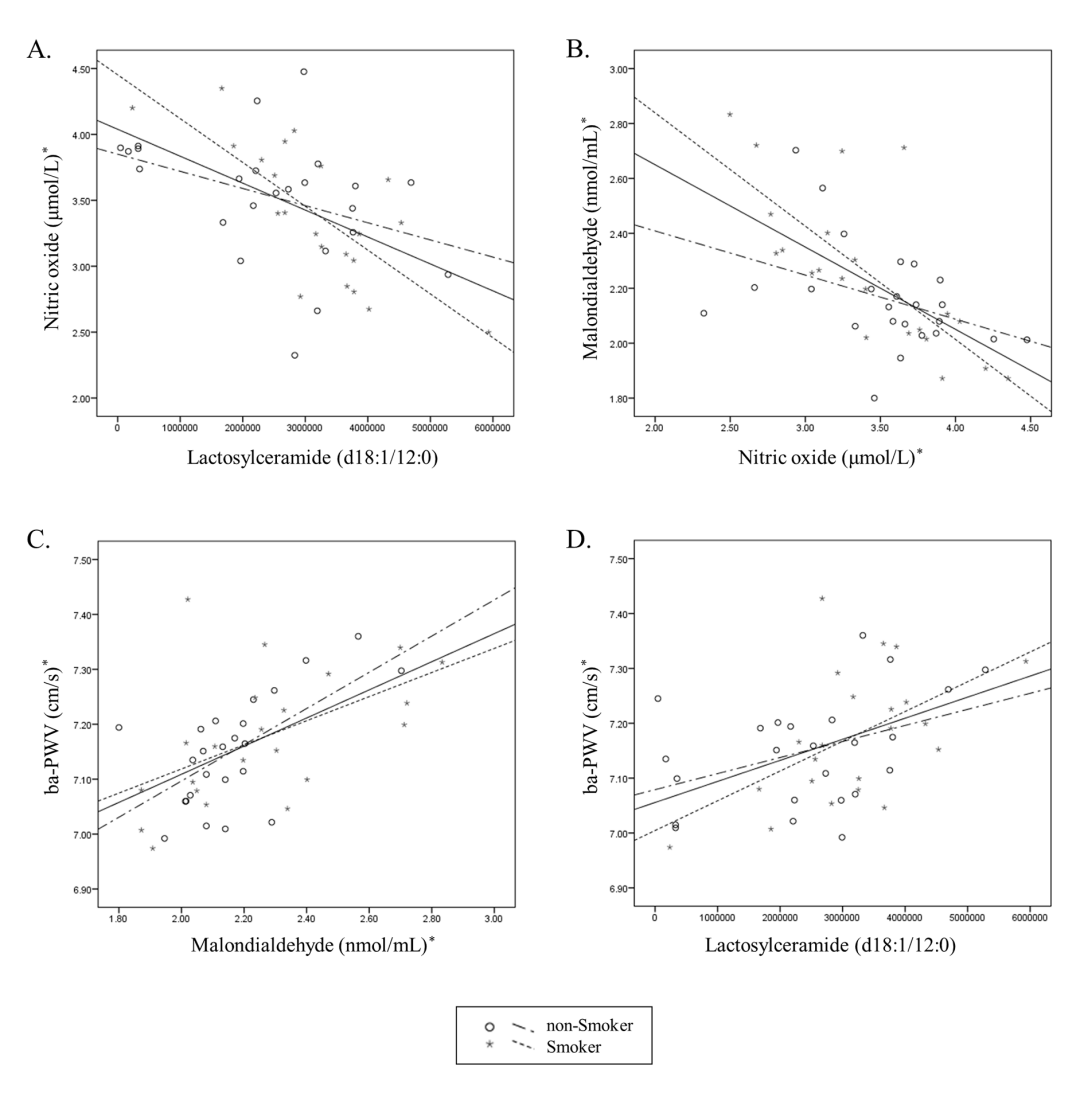
Statistical relations of lactosylceramide, nitric oxide, malondialdehyde, and ba-PWV. Relationship of lactosylceramide (d18:1/12:0), nitric oxide, malondialdehyde, and ba-PWV in male subjects under 50 yrs.^*^Tested by log-transformed. Tested by Pearson correlation (*r*_*0*_: smoker, *r*_*1*_: non-smoker, *r*_*2*_: total). (A) *r*_*0*_ = -0.739, *P*_*0*_<0.001; *r*_*1*_ = -0.388, *P*_*1*_ = 0.061; *r*_*2*_ = -0.551, *P*_*2*_<0.001. (B) *r*_*0*_ = -0.751, *P*_*0*_<0.001; *r*_*1*_ = -0.400, *P*_*1*_ = 0.053; *r*_*2*_ = -0.612, *P*_*2*_<0.001. (C) *r*_*0*_ = 0.526, *P*_*0*_ = 0.012; *r*_*1*_ = 0.628, *P*_*1*_ = 0.001; *r*_*2*_ = 0.570, *P*_*2*_<0.001. (D) *r*_*0*_ = 0.527, *P*_*0*_ = 0.012; *r*_*1*_ = 0.414, *P*_*1*_ = 0.044; *r*_*2*_ = 0.470, *P*_*2*_ = 0.001.

**Fig 6:**
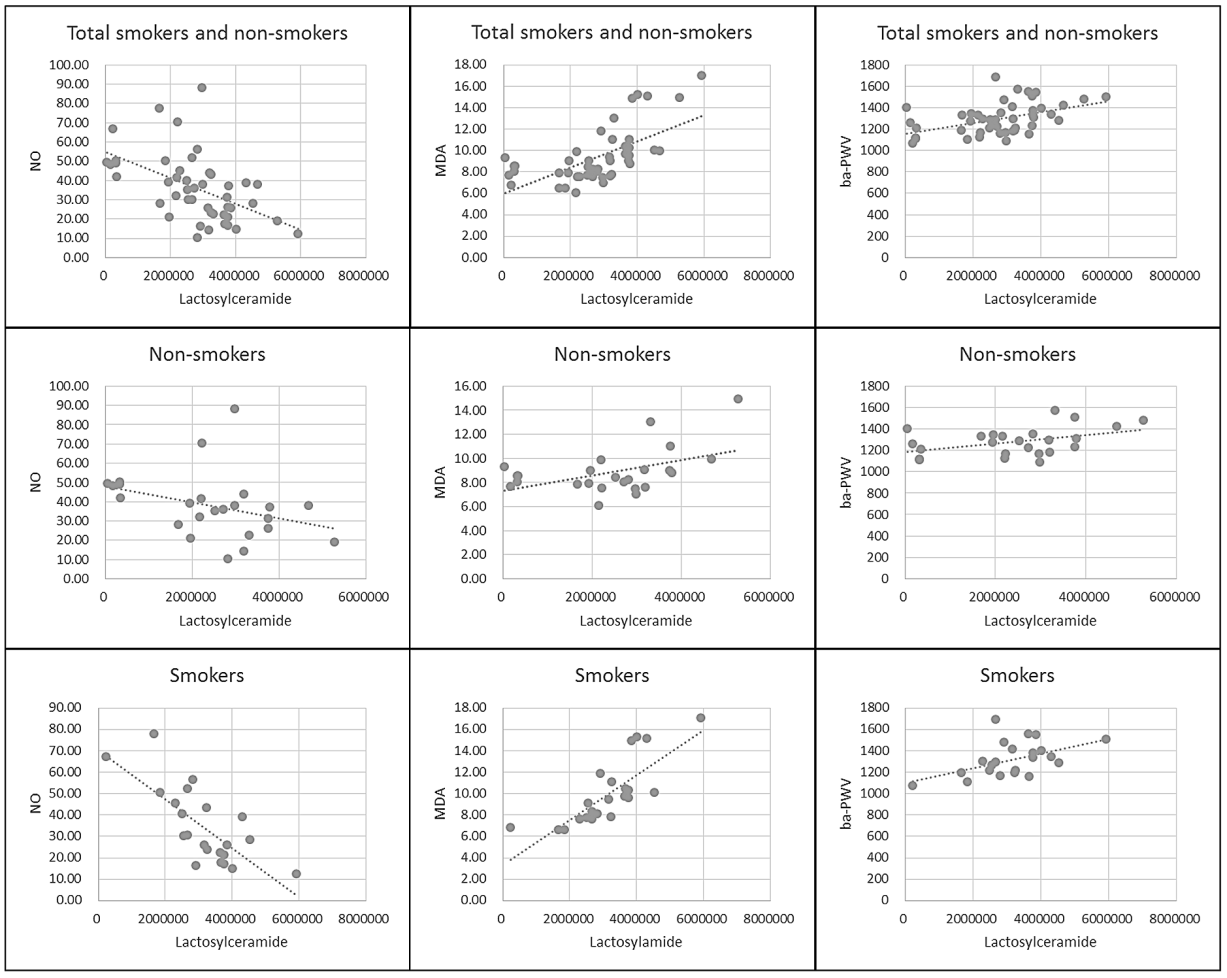
Overall view of nitric oxide, malondialdehyde, and ba-PWV with lactosylceramade.

The experiments showed that there were no differences in biochemical characteristics between smoker and non-smoker subjects (Table 4). In correlation analysis (Fig 5), lactosylceramide correlated negatively with NO and NO correlated negatively with plasma MDA. Additionally, MDA positively correlated with ba-PWV (Brachial-ankle pulse wave velocity) which is a marker for arterial stiffness. Finally, lactosylceramide correlated positively with ba-PWV. To understand the flow from lactosylceramide to arterial stiffness more clearly, we investigated the relationships among the four entities- lactosylceramide, NO, MDA and arterial stiffness (which is shown by ba-PWV) under the condition of fixing lactosylceramide on the x-axis (Fig 6). Overall through our results we ascertained that the subjects with low levels of lactosylceramide tend to have relatively high NO levels and low MDA levels, followed by lower ba-PWV while subjects with high levels of lactosylceramide tend to have relatively low NO levels and high MDA levels, followed by higher ba-PWV. This was evident for both smokers and non-smokers while the trend was more powerful in the smoker group. This being so, we could suggest that lactosylceramide, NO, MDA, and arterial stiffness are closely related, and that the interrelation between lactosylcermiade, NO and MDA might have an impact on arterial stiffness, though further study is needed to investigate the chain mechanism.

**Table 4:** Clinical and biochemical characteristics in male subjects under 50 yrs.

	Smoker (*n* = 22)	Non-smoker (*n* = 24)	Total (*n* = 46)	*P*-value
Age (year)	39.7±0.92	41.4±0.90	40.6±0.65	0.284
Body mass index (kg/m^2^)	23.7±0.51	23.9±0.47	23.8±0.34	0.982
Systolic BP (mmHg)	121.0±2.22	120.6±2.23	120.8±1.56	0.982
Diastolic BP (mmHg)	75.2±1.83	76.5±1.96	75.9±1.33	0.676
Triglyceride (mg/dL)^*∮*^	127.6±9.11	116.0±14.8	121.5±8.83	0.113
Total-cholesterol (mg/dL)^*∮*^	179.6±7.04	193.3±7.45	186.7±5.19	0.132
HDL-cholesterol (mg/dL)^*∮*^	50.3±1.87	54.5±3.35	52.5±1.97	0.454
LDL-cholesterol (mg/dL)^*∮*^	103.8±6.75	115.6±6.51	110.0±4.72	0.111
Glucose (mg/dL)^*∮*^	89.8±2.40	91.8±2.01	90.9±1.54	0.366
Insulin (μIU/dL)^*∮*^	8.31±0.69	9.35±0.84	8.85±0.55	0.317
Malondialdehyde (nmol/mL)^*∮*^	9.98±0.65	8.86±0.39	9.39±0.38	0.253
Nitric oxide (μmol/L)^*∮*^	34.1±3.81	37.8±3.48	36.1±2.56	0.410
ba-PWV (cm/s)^*∮*^	1316.2±34.0	1280.8±26.7	1297.7±21.3	0.575
Lactosylceramide (d18:1/12:0)	3156288±246573	2437072±290662	2781045±197400	0.086

Mean ± SE.

^∮^tested by logarithmic transformation, *P*-values derived from independent *t*-test between smoker and non-smoker.

**Fig 7:**
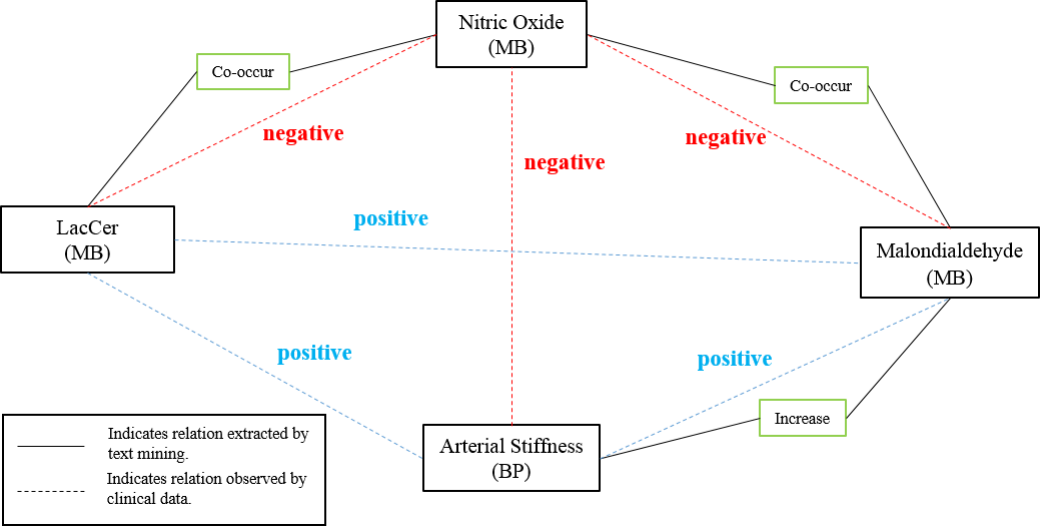
Network relations of lactosylceramade, nitric oxide, malondialdehyde, and arterial stiffness.

We specifically confirmed a strongly negative correlation between total NO and plasma MDA, a marker of oxygen-derived free radicals, was consistent with a previous report [42]. Bioavailability of NO is a critical factor to maintain normal vascular functions including vasomotor reactivity, anti-thrombosis state, barrier function and non-adhesive state to inflammation cells [43]. Kim et al. [44] reported that the changes in ba-PWV (a marker of arterial stiffness) [45] were positively correlated with changes in MDA which was coincide with present validated data. In the endothelium, endothelial nitric synthase (eNOS) converts L-arginine to L-citrulline and NO. Active NO levels are largely regulated by eNOS gene expression or its activity [46]. Many cardiovascular risk factors could negatively affect NO levels by different mechanisms, thus, present data has shown lactosylceramide correlated negatively with NO. However, it is not clear whether lactosylceramide could affect vasomotor reaction and eNOS expression. Through the confirmed relationships among lactosylceramide, NO, MDA, and ba-PWV, therefore, the correlation between lactosylceramide, NO and MDA could reflect early adverse vascular changes and precede lipid peroxides. In addition, we could partly suggest those three biomarkers, lactosylceramide, NO, and MDA, may closely link to arterial stiffness. It is important to notice that our results do not imply a chain mechanism, in which increased lactosylceramide represses NO, resulting in increased levels of MDA, which in the end results in increased arterial stiffness, but rather shows, a net-work effect in which the interrelation between lactosylcermiade, NO and MDA might have an impact on arterial stiffness (Fig 7).

However, Although data is not shown, regression analysis showed a positive relation between lactosylceramide and MDA, which means lactosylceramide was emerged as an independent predictor of MDA (p<0.05), a negative relation between MDA and NO which means MDA was emerged as an independent predictor of NO (p<0.05), and a negative relation between NO and ba-PWV which means NO was emerged as an independent predictor of ba-PWV (p<0.05), while other relations were not significant. This suggests a chain mechanism in which increased lactosylceramide will result in increased MDA, which represses NO, and in the end will result in increased ba-PWV, however, further research is needed to confirm our findings.

## Discussion

The field of LBD is experiencing substantial growth in recent years as databases, ontologies, and text mining tools are actively and competitively being developed. Especially, text mining techniques such as NER, event extraction, or dependency parser enable mining more plentiful information and knowledge with a wider diversity from the academic literature, whose amount is too heavy to be manually handled and digested. Investigators have indeed attempted to find not only the hidden relationships between different pairs of entity types like protein-disease or drug-disease [2,8,9], but also biomarkers [10], and drug indications [11]. One example is that Vos et al. derived new plausible multimorbidity patterns of psychiatric and somatic diseases using automated concept recognition and profiling [12]. But they studied only the pairwise associations of diseases, distinguishing itself from our study in terms of the limited scope and number of biological entities within one hypothesis.

Unlike previous studies, our method proposes the extended ABC model that acts as the theoretical background contains multiple steps of B terms while other studies keep solely one step [2–6]. Each candidate in Table 2 involves from one up to three B terms. This eventually leads to the broader view on the relationships among multiple biological concepts, connecting lactosylceramide, NO, MDA, and arterial stiffness all together. It also enhances the variety and the concreteness of candidates for the developed hypothesis, constituting information flow. Moreover, the information flow discovered in our study is supported by the type and directionality of the relationship which was seldom provided in the related works despite its significance. Our approach is also differentiated from the study on integrated bio-entity network by Bell et al. [7] in that our approach automatically extracts the relationship information from the literature, surpassing the limitation of referencing the databases and opening up a higher possibility for novel discovery.

The experiments show that the proposed literature mining-based approach helps develop and enrich the existing hypothesis, detecting the previously unrevealed relationship between lactosylceramide, NO, MDA, and arterial stiffness. While the associations of some two of them were already verified in the earlier studies, our study was the first to draw the comprehensive picture of all four of the biomedical entities. Our method also successfully offered the context for the developed hypothesis, which is crucial for hypothesis enrichment and storytelling. In the literature mining experiment the positive correlation between MDA and arterial stiffness manifested with an aid of the contextual information (the *Increase* relationship between them) for supporting the tested hypothesis. The clinical experiment assured the credibility and practicability of the method.

When it comes to the algorithmic side, we uniquely adopted the concept of semantic relatedness as the criterion for ranking the developed hypothesis candidates that consist of more than two entity pairs. Hypothesis ranking is crucial to handle the issue of false positives and retain the usefulness of the method [7]. We conducted statistical analysis to examine how much the semantic relatedness is reliable for determining the relationship of an entity pair. Among various combinations of entity types, we selected one of the most frequently studied entity relation, gene-disease relation. To the best of our knowledge, DisGeNET [47] is the only database which provides with the credibility score of entity-entity association. We could obtain the scores of gene-disease associations from DisGeNET, which take into account only curated databases. Our data had a total of 1,041 gene-disease pairs with each semantic relatedness score. After an automatic matching partly supplemented by a manual curation, we found the final 45 pairs had both semantic and database-based relatedness score. Testing on those 45 pairs, Pearson correlation coefficient was calculated with the two types of scores.

**Fig 8:**
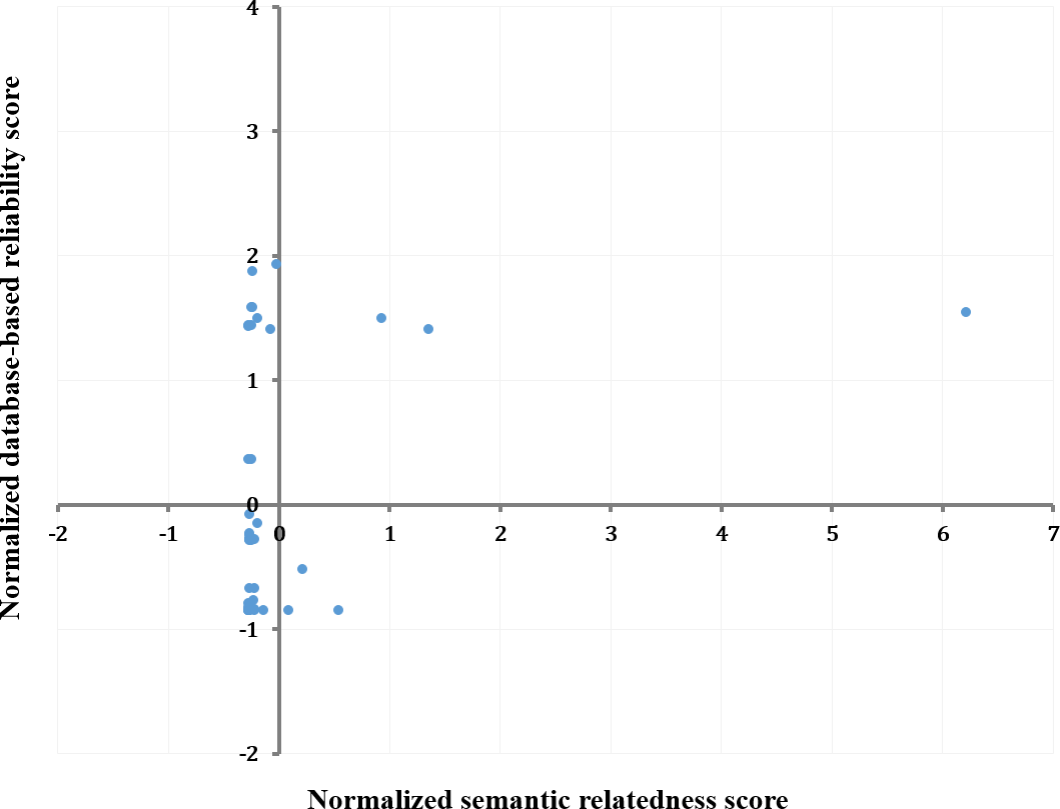
Scatterplot of database-based versus semantic relatedness score (both normalized).

The correlation between semantic and database-based relatedness score was statistically significant (*r* = 0.31, *P*<0.05). In other words, database-based relatedness score tends to increase to some degree as more semantic relatedness score is given for a gene-disease pair (Fig 8). This means that the semantic relatedness derived from the unstructured text can act at least a supplementary means to discover the biomedical associations that have already been or will be included in the curated, structured sources. Accordingly, we could verify the validity of the ranking algorithm we applied.

Lastly, our study extends to involve the clinical observations so that we can test if the proposed methodology can play a crucial role in the biological research. Many of earlier studies have not examined the practical usefulness of literature mining for knowledge discovery [4–12]. In this regard, the study well demonstrates the characteristic and the value of bio text mining at the intersection of biology and information technology. Our proposed method can be regarded as an effective means to promote knowledge discovery in the biomedical field. We expect our proposed method would conduce to the research in the biological field by supporting and elaborating researchers’ hypotheses, or predicting the logical process within them.

## Conclusions

The automatic generation of plausible new hypotheses is a daunting challenge specifically when multiple entities and relationships are interconnected at different levels. In addition, the confirmation step of generated hypotheses ought to be considered to make such a difficult, complicated task of new hypothesis meaningful. To this end, we have presented the new method for new hypothesis development and enrichment, which helps biologists extend their hypotheses or explain a logical process within the validated hypotheses. The method is developed by integrating state-of-the-art text mining techniques and a unique measure of ranking score, which is differentiated from the existing similar systems. We demonstrated how the method can be applied to elaborate on the specific metabolite-related hypothesis. As a result, we found that the proposed method is reliable and practically applicable to the biomedical field through the experiments the domain experts are involved in.

The major limitation of the present study is that we only verified one hypothesis. To make the proposed approach more reliable, we plan to conduct more clinical experiments for other plausible hypotheses generated by the proposed approach. Another limitation lies in extracting entities and relations. We plan to implement a named entity disambiguation technique to resolve the weakness of dictionary-based NER and to develop an event extraction technique for producing a richer context.

## Supporting information

S1 Table Top ranked candidates of the developed hypothesis using BITOLA. (TIF)

S2 Table Top ranked candidates of the developed hypothesis using SemRep. (TIF)

## Abbreviations

LBD: literature-based discovery
CVD: cardiovascular disease
ba-PWV: brachial-ankle PWV
NER: named entity recognition
MeSH: medical subject headings
RE: relation extraction
COALS: correlated occurrence analogue to lexical semantic
MB: metabolite
GE: gene/protein
BP: biological process/pathway
DS: disease
BD: body/organ
LacCer: lactosylceramide
NO: nitric oxide
MDA: malondialdehyde
eNOS: endothelial nitric synthase
