# Supplementary figures and images for: Enriching plausible new hypothesis generation in PubMed
Source: PLoS One. 2017 Jul 5;12(7):e0180539. doi: 10.1371/journal.pone.0180539 (PMC5498031; doi:10.1371/journal.pone.0180539)

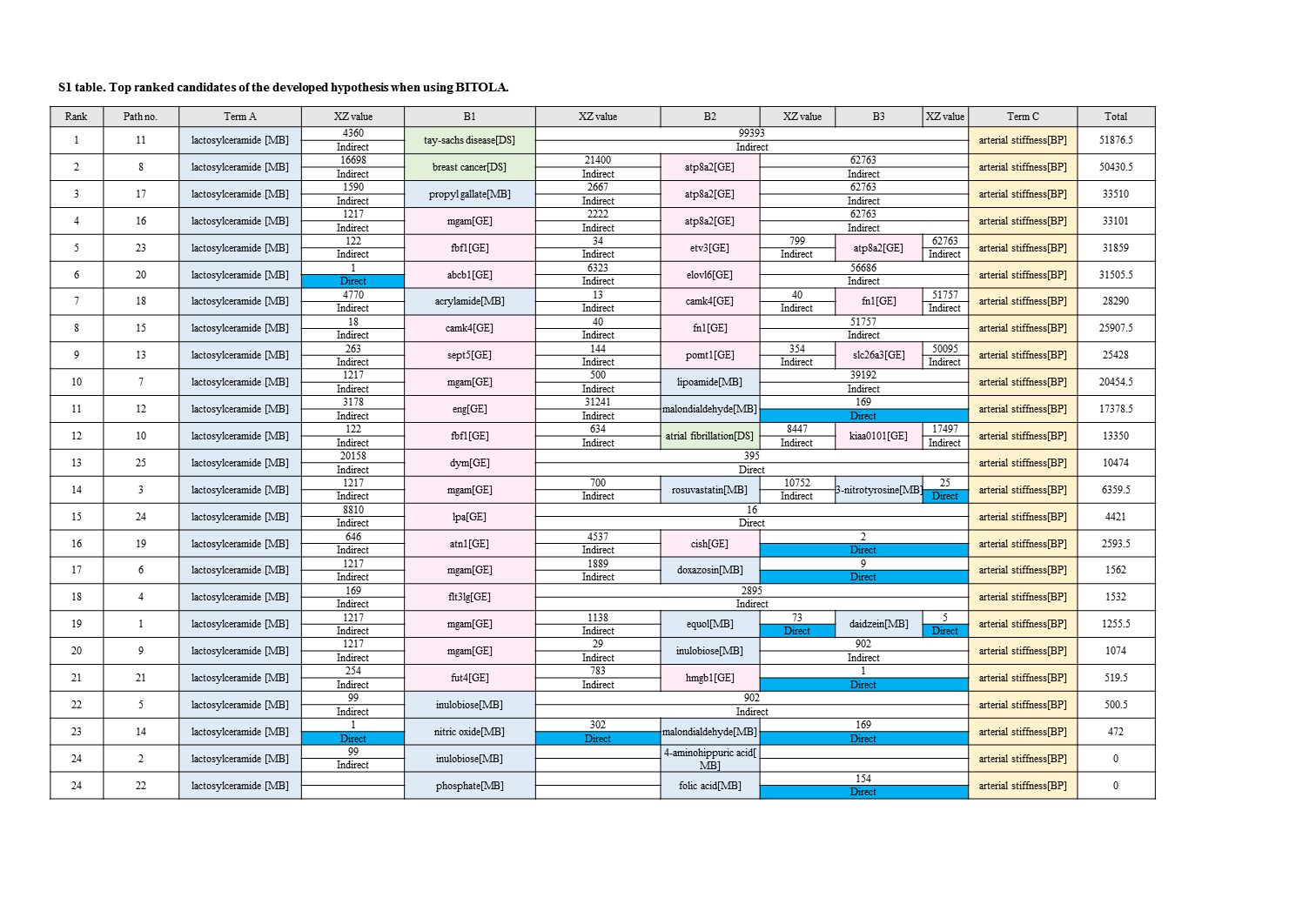

Supplement: S1 Table — (TIF) [file pone.0180539.s001.tif]

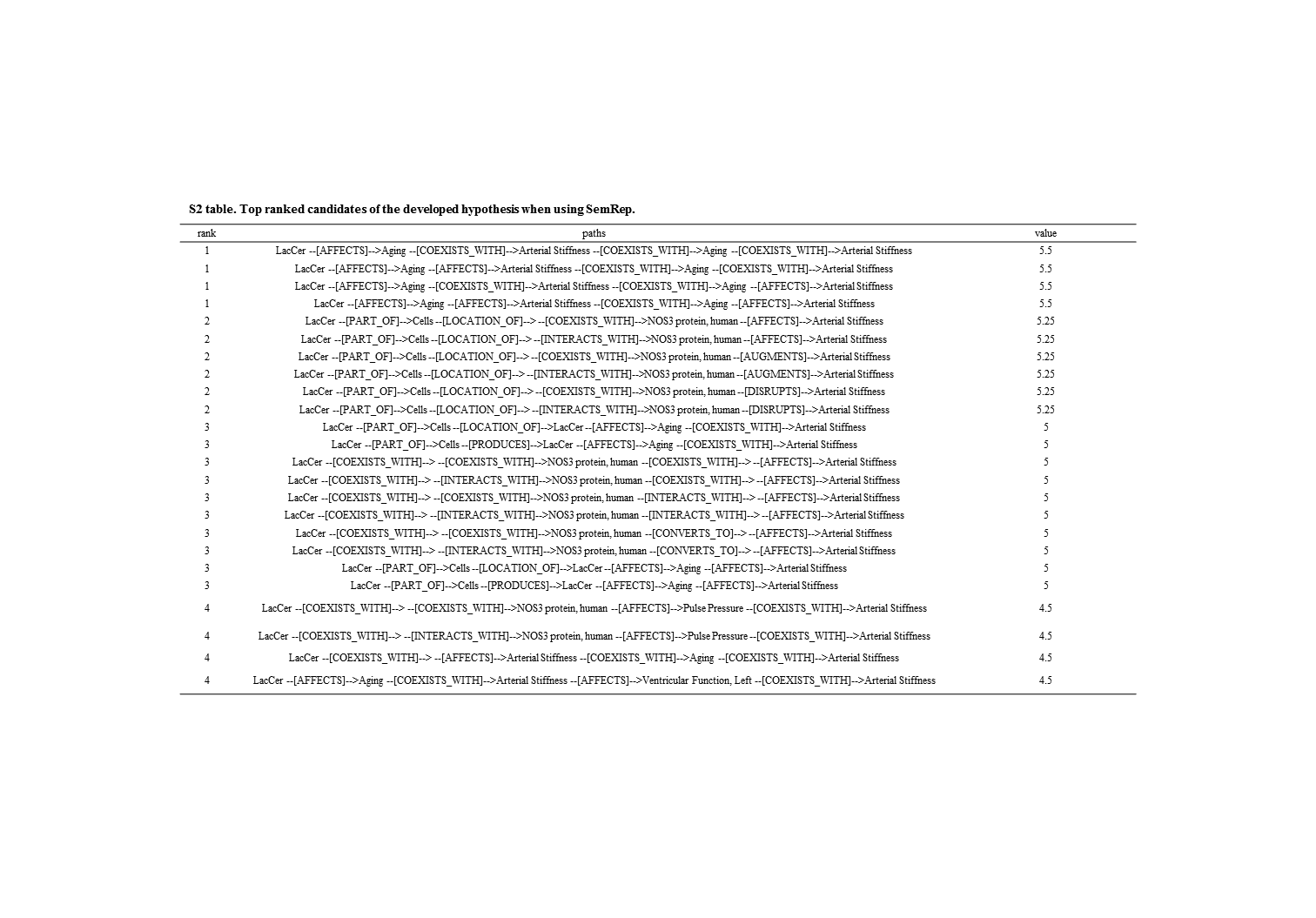

Supplement: S2 Table — (TIF) [file pone.0180539.s002.tif]
